# Gellan Gum/Laponite Beads for the Modified Release of Drugs: Experimental and Modeling Study of Gastrointestinal Release

**DOI:** 10.3390/pharmaceutics11040187

**Published:** 2019-04-17

**Authors:** Alessandra Adrover, Patrizia Paolicelli, Stefania Petralito, Laura Di Muzio, Jordan Trilli, Stefania Cesa, Ingunn Tho, Maria Antonietta Casadei

**Affiliations:** 1 Dipartimento di Ingegneria Chimica, Materiali e Ambiente, Sapienza Universitá di Roma, Via Eudossiana 18, 00184 Rome, Italy; 2 Dipartimento di Chimica e Tecnologie del Farmaco, Sapienza Universitá di Roma, Piazzale Aldo Moro 5, 00185 Rome, Italy; patrizia.paolicelli@uniroma1.it (P.P.); stefania.petralito@uniroma1.it (S.P.); laura.dimuzio@uniroma1.it (L.D.M.); jordan.trilli@uniroma1.it (J.T.); stefania.cesa@uniroma1.it (S.C.); mariaantonietta.casadei@uniroma1.it (M.A.C.); 3 Department of Pharmacy, University of Oslo, 0316 Oslo, Norway; ingunn.tho@farmasi.uio.no

**Keywords:** beads, gellan gum, ionotropic gelation, laponite, modeling study, swelling, gastrointestinal drug release, polymer/clay composite

## Abstract

In this study, gellan gum (GG), a natural polysaccharide, was used to fabricate spherical porous beads suitable as sustained drug delivery systems for oral administration. GG was cross-linked with calcium ions to prepare polymeric beads. Rheological studies and preliminary experiments of beads preparation allowed to identify the GG and the CaCl_2_ concentrations suitable for obtaining stable and spherical particles. GG beads were formed, through ionotropic gelation technique, with and without the presence of the synthetic clay laponite. The resultant beads were analyzed for dimensions (before and after freeze-drying), morphological aspects and ability to swell in different media miming biological fluids, namely SGF (Simulated Gastric Fluid, HCl 0.1 M) and SIF (Simulated Intestinal Fluid, phosphate buffer, 0.044 M, pH 7.4). The swelling degree was lower in SGF than in SIF and further reduced in the presence of laponite. The GG and GG-layered silicate composite beads were loaded with two model drugs having different molecular weight, namely theophylline and cyanocobalamin (vitamin B12) and subjected to in-vitro release studies in SGF and SIF. The presence of laponite in the bead formulation increased the drug entrapment efficiency and slowed-down the release kinetics of both drugs in the gastric environment. A moving-boundary swelling model with “diffuse” glassy-rubbery interface was proposed in order to describe the swelling behavior of porous freeze-dried beads. Consistently with the swelling model adopted, two moving-boundary drug release models were developed to interpret release data from highly porous beads of different drugs: drug molecules, e.g., theophylline, that exhibit a typical Fickian behavior of release curves and drugs, such as vitamin B12, whose release curves are affected by the physical/chemical interaction of the drug with the polymer/clay complex. Theoretical results support the experimental observations, thus confirming that laponite may be an effective additive for fabricating sustained drug delivery systems.

## 1. Introduction

Orally administered dosage forms are the most convenient formulations due to the easiness of employment, pre-determined and measured doses and overall non-invasive nature of administration, which increase the patient compliance. The successful oral formulation should deliver the required therapeutic dose to the specific site of action during the treatment period. However, the delivery of a drug by a simple conventional dosage form normally results in the immediate release of the active pharmaceutic ingredient and their use usually requires a high frequency of administration and uncontrolled absorption. These considerations have guided researchers to focus their efforts on improving oral delivery systems with the development of formulations providing more predictable release rates as well as an increased bioavailability. Sustained release formulations are extensively investigated in order to reduce the dosing frequency, thus resulting in increased patience compliance.

In the last decades, many biomaterials have been proposed as interesting materials in the design of modified oral drug delivery systems in order to accomplish therapeutic or convenience purposes not offered by conventional dosage forms. 

Synthetic and natural polymers have been proposed for the development of oral extended-release dosage forms. However, naturally derived polymers offer many advantages over synthetic polymers related to their biocompatibility, biodegradability, non-toxicity and reasonable costs. 

In particular, hydrophilic polymers, such as polysaccharides, are widely used as natural materials in sustained oral dosage forms and the interest in the application of these polymers for prolonging drug release has increased over the last decades [1]. The interest toward these polymers is related to their swelling and filmogen [2] capabilities as well as their pH-sensitive [3,4] or floating capability [5,6,7,8]. 

Natural gums, such as gellan gum (GG), an anionic, high molecular weight polysaccharide, has gained significant interest in the pharmaceutical field [9]. It consists of tetra-saccharide repeating units: α-l-rhamnose, β-d-glucuronic acid and β-d-glucose in the molar ratio 1:1:2. 

GG has proven to be a versatile material in the formulation of polymeric hydrogels, including beads systems, due to its temperature sensitivity and ability to gel under mild conditions. In fact, it forms stable hydrogel networks in the presence of cationic cross-linkers [10,11], so that ionotropic gelation method can be employed for the synthesis of polymeric networks using divalent cations as cross-linking agents [12,13,14]. The contact of the polymer with cations results in the instantaneous formation of a gel matrix containing uniformly dispersed material throughout the crosslinked gellan gum matrix. 

Polymeric beads are widely used for oral sustained release; after beads are ingested, the drug will slowly diffuse out from the polymer matrix, resulting in a prolonged release of the active agent. Nevertheless, some drawbacks, related to the higher porosity of the matrix or poor mechanical resistance of the polymeric network, could lead to a rapid and massive release in acidic dissolution medium [15]. Only a few polymers can be used in their pure form for the formulation of oral sustained release beads and therefore their combination with other biocompatible materials has been investigated in order to overcome these drawbacks. Clay minerals are one of the fillers that can be used, in combination with many biopolymers, to improve their drug delivery properties [16,17,18,19]. The ultimate goal is to bring together in the same material the best properties of the natural polymer and clay since each component plays a key role in improving the properties of the nanocomposite hydrogels.

In this scenario, clay hydrogel beads have been widely investigated in oral drug delivery applications, showing that mineral clays can be successfully used as functional additives in the development of bead-modified systems [20]. 

The most commonly used clay minerals belong to the smectite family. Among the smectite family, laponite Na_0.7_[(Si_8_Mg_5.5_Li_0.3_)O_20_(OH)_4_]_0.7_ is a synthetic clay composed of a layered structure (30–25 nm diameter, 1 nm thickness) that has been used to synthesize a wide range of nano-composite hydrogels [21,22,23]. Specifically, laponite (LAPO) nanoparticles can be uniformly dispersed within the polymeric matrix where they self-arrange and act as both filler and cross-linker during gel formation [24,25]. 

This study aims to verify the possibility of using laponite as an additive clay mineral to design new composite gellan gum beads with highly specific characteristics, such as appropriate swelling properties and release kinetics.

Rheological studies and preliminary experiments of bead preparation allowed selecting the gellan gum and crosslinker (CaCl_2_) concentrations suitable for obtaining stable and spherical particles. Under optimized experimental conditions, laponite was uniformly dispersed in the polymeric solution allowing the formation of nano-composite GG beads with reduced mesh size. In order to investigate how the morphology, swelling and the release properties of the nano-composite hydrogels were affected by the laponite, beads were loaded with two model drugs having different molecular weights and release studies were performed in simulated gastric fluid (SGF) and in simulated intestinal one (SIF). Mathematical models for swelling and drug release from these highly porous beads were proposed. Reliable values of drug diffusion coefficients in different release media were obtained. 

## 2. Materials and Methods

### 2.1. Chemicals

Theophylline, vitamin B12, methanol, acetic acid, hydrochloric acid and low acyl gellan gum (Gelzan^TM^) were purchased from Sigma Aldrich Company (Darmstadt, Germany), and calcium chloride hydrate, potassium dihydrogen phosphate and sodium hydroxide from Carlo Erba Reagents S.r.l (Milan, Italy). We used bidistilled water from Carlo Erba Reagents S.r.l. for the HPLC analysis. For sample preparation and all the other analyses, we used demineralized water produced with a Pharma20 equipment, Culligan Italiana S.p.A (Bologna, Italy). Laponite XLG was a gift of Rockwood Additives Ltd. (Moosburg, Germany). 

### 2.2. Rheological Measurements

Rheological experiments were performed with a Haake RheoStress 300 Rotational Rheometer (Dreieich, Germany) equipped with a Haake DC10 thermostat. Oscillatory experiments were performed at 25.0 ± 0.2 °C in the range 0.01–10 Hz on the hydrogels obtained from 1.0, 1.5 and 2.0% *w*/*w* GG solutions. Enough quantity of each sample was carefully poured to completely cover the 6 cm cone-plate geometry (angle of 1°). For each sample, the linear viscoelastic range was evaluated: a 1% maximum deformation was used. 

### 2.3. Beads Preparation 

Gellan gum (0.11, 0.165 or 0.22 g) was added to 11 mL of double distilled water and maintained under stirring for 5 h at 80 °C until a homogeneous solution was produced. This solution was cooled and kept at 40 °C. Then, 10 mL of this solution were carefully loaded into a syringe with a 21G needle, ensuring no air bubbles were present, and added to a solution of calcium chloride (50 mL, 0.3% and 0.6% *w*/*w*) drop wise. The beads were left cross-linking for 10 min (curing time), then filtered and washed four times with 10 mL of deionized water and freeze-dried. The curing time was optimized to have maximum entrapment efficiency of the model molecules used. In fact, while longer curing times increase the degree of crosslinking of the polymer, they also promote the effusion of the loaded molecule out of the beads, thus reducing the final drug loading.

Beads including laponite were produced starting from a solution (11 mL) of GG (1.5% *w*/*w*) and laponite (1.0% *w*/*w*) added drop wise to the solution of CaCl_2_ (0.3% *w*/*w*), thus following the same procedure adopted for beads without laponite. 

The diameter of fresh and freeze-dried beads was measured with a caliper along two orthogonal directions, taking the average of the measurements as the mean diameter of the beads, whereas the ratio between the two measurements was taken as the aspect ratio of the beads. 

### 2.4. Determination of Swelling Degree 

In order to quantify the swelling degree of the beads, 10 freeze-dried beads were weighed and placed into a tulle net and submerged into 25 mL of simulated gastric fluid (SGF, HCl 0.1 M) or simulated intestinal fluid (SIF, phosphate buffer 0.044 M, pH 7.4), maintained at 37.0 ± 0.5 °C. After 5 min, the beads were removed, lightly blotted on paper to remove the excess liquid and weighed. The beads were then submerged back into the medium and the process was repeated at established time intervals up to 24 h. The experiments were carried out in triplicate with each value representing the mean ± SD. 

The swelling degree *S* was calculated using the following equation:

(1) $$\;S=\frac{weight\;of\;swollen\;beads\;-\;weight\;of\;dry\;beads\;}{weight\;of\;dry\;beads\;}.$$

### 2.5. Preparation of Drug Loaded Beads 

Gellan gum (0.165 g) was dissolved in 9 mL of distilled water using the method described in Section 2.3. Theophylline or vitamin B12 (0.0146 g) were solubilized in 2 mL of water and added to the cooled gellan gum solution, to make a final volume of 11 mL and a concentration of 1.5% *w*/*w* of GG. The solution was stirred at 100 rpm for 10 min to ensure the drug homogeneously dispersed. The beads were then formed using the method described in Section 2.3. Drug loaded beads including laponite were prepared from a starting solution (9 mL) of gellan gum (0.165 g) and laponite (0.11 g) and then following the same procedure adopted for drug loaded beads without laponite.

### 2.6. Drug Entrapment Efficiency

In order to determine the quantity of drug loaded into the beads, 15 mg of freeze-dried beads were stirred vigorously in SIF for 1 h, to destroy the beads and extract the drug. The solution was filtered and analyzed by HPLC. The apparatus consisted of a Perkin Elmer Series 200 LC pump, equipped with a 235 Diode Array Detector and a Total-Chrom data processor (Perkin Elmer, Waltham, MA, USA). HPLC analyses were carried out using a Merck Hibar LiChrocart (250–4.5 μm) RP-18 column under isocratic conditions (0.7 mL/min) using a mobile phase constituted by methanol and acetic acid (0.1 M) mixture in a proportion of 40:60 (*v*:*v*). Theophylline was monitored at λ = 280 nm and vitamin B12 at λ = 360 nm. Under these conditions, the retention time of theophylline was about 6 min, while that of vitamin B12 was about 4 min. 

The drug entrapment efficiency was calculated using the following equation:

(2) $$Drug\;entrapment\;efficiency\;(\%)\;=\;\frac{actual\;drug\;content\;of\;beads}{theoretical\;drug\;content\;of\;beads}\;\times \;100.$$

All experiments were carried out in triplicate and each value reported representing the mean, ± SD. 

### 2.7. In Vitro Release Studies

Release studies from drug loaded beads with different formulations were performed separately in SGF (HCl 0.1 M) and in SIF (phosphate buffer, pH 7.4) and sequentially in SGF and SIF to simulate the drug release in the entire gastrointestinal tract. A total of 15 mg of drug loaded beads were added to a known volume *V_res_* of SIF or SGF, warmed to 37 °C in a water bath and stirred continuously at 200 rpm. At defined times, from 1 to 240 min, 1 mL of solution was withdrawn and replaced with 1 mL of fresh solution. Different volumes *V_res_* = 50, 75, 100, 150, 175 mL were considered in order to investigate the influence of the release volume *V_res_* on release curves. See Section 4.5.1 for a discussion on the role of *V_res_*.

For gastrointestinal in-vitro release experiments, 15 mg of drug-loaded beads were added to 100 mL of SGF, warmed to 37 °C in a water bath and stirred continuously at 200 rpm. At defined times, from 1 to 120 min, 1 mL of solution was withdrawn and replaced with 1 mL of fresh SGF. After 120 min, the beads were drained to remove excess acid and transferred into 50 mL of SIF. Every 15 min, 1 mL of solution was withdrawn and replaced with the same volume of SIF until 240 min and then again after 24 h. By considering that, after the first 120 min, the beads had released from 60% to 95% of the initially loaded drug, depending on the bead formulation, we chose to carry out the subsequent release in SIF in a smaller release volume (half of that in SGF) to maintain the drug concentration in the release volume high enough to allow the subsequent HPLC analysis. Drug concentrations were determined by HPLC analysis as reported in the previous section. 

After 24 h, the beads were collected from the media and destroyed to extract and quantify the drug still embedded into the beads. The release data were reported as drug concentration *C*_res_(*t_w_^i^*) [mg/mL] at withdrawal times *t_w_^i^* [min] and as fraction of drug released up to time t_w_^i^ with respect to the total amount of drug loaded in the beads. The experiments were carried out in triplicate with each value reported representing the mean ± SD. 

### 2.8. Statistics

Statistical tests were performed to evaluate the effect of laponite on gastrointestinal release rates of the two model molecules. Statistical analysis was performed with GraphPad Prism™ (Version 4.00) software (GraphPad Software, Inc., San Diego, CA, USA). The Student’s *t*-test was applied to determine the statistical significance between two different experimental conditions. The values of *p* < 0.05 were considered significant.

## 3. Mathematical Modeling of Swelling and Drug release of Highly Porous Beads

### 3.1. Swelling Modeling

**Figure 1:**
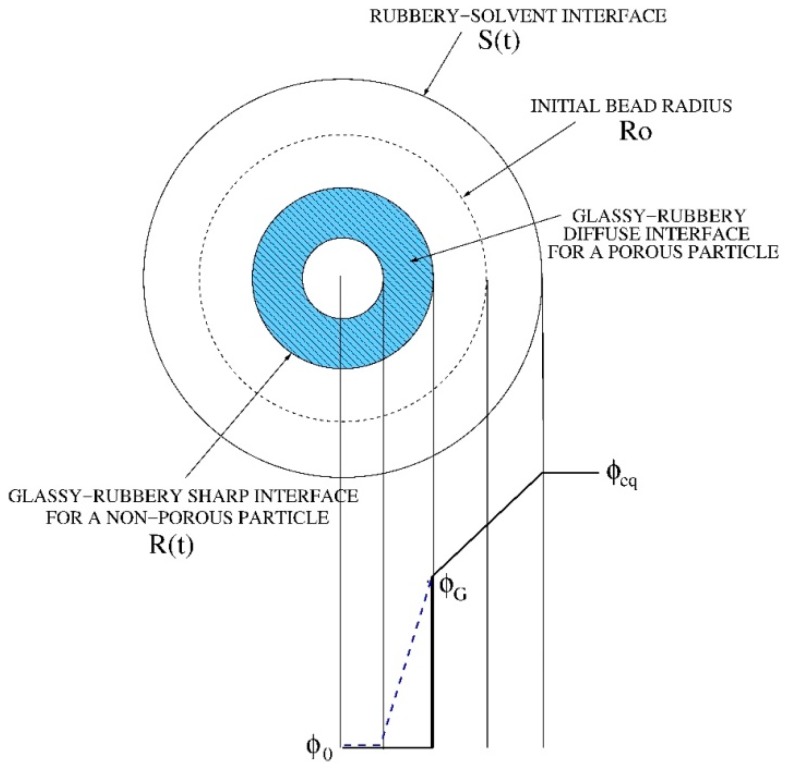
Schematic representation of moving fronts and solvent concentration profiles in a swelling process of a dry spherical bead. The two cases of a non-porous and a highly porous particle (blue dotted line) are shown.

We adopted a radial one-dimensional model of swelling of spherical dry beads. A classical approach to swelling of glassy polymers requires the solution of a moving boundary model describing the solvent transport in the swollen gel and the time evolution of two fronts [26,27,28], the erosion front (rubbery-solvent interface at *r = S(t)*) and the swelling front (glassy-rubbery interface at *r = R(t)*), as shown in Figure 1. 

The solvent balance equation, written in terms of the solvent volumetric fraction *φ(r,t)* reads

(3) $$\frac{\partial \varphi }{\partial t}=\frac{1}{r^{2}}\frac{\partial }{\partial r}\left(r^{2}D_{s}\left(\varphi \right)\frac{\partial \varphi }{\partial r}-r^{2}v_{r}^{s}\left(\varphi \right)\varphi \right)=\frac{1}{r^{2}}\frac{\partial }{\partial r}\left(r^{2}D_{s}\left(\varphi \right)\left(1-\varphi \right)\frac{\partial \varphi }{\partial r}\right),\;R\left(t\right)<r<S\left(t\right),$$

where *v_r_^s^(φ)* is the swelling velocity *v_r_^s^(φ) = D_s_(φ)∂φ/∂r* and *D_s_(φ)* is the solvent diffusion coefficient, that can be assumed constant or a function of the solvent volume fraction *φ*. 

Equation (3) must be solved with the boundary conditions,

(4) $${\left[\varphi \right]}_{r=S\left(t\right)}=\varphi_{eq},{\left[\varphi \right]}_{r=R\left(t\right)}=\varphi_{G}\;for\;R\left(t\right)>0,{\left[\frac{\partial \varphi }{\partial r}\right]}_{r=0}=0\;for\;R\left(t\right)=0,$$

and initial conditions *φ*(*r*,0) *= φ_0_* for 0 *≤ r ≤ R*_0_, where *R*_0_ is the initial radius of the dry particle, *φ_eq_* is the volume fraction at equilibrium and *φ_G_* is the threshold volume fraction to initiate swelling.

If we assume that the glassy phase for *r < R(t)* is totally impermeable to the solvent (the solvent diffusivity is zero in the glassy phase) the two fronts, the glassy-rubbery at *r = R(t)* and the rubbery-solvent at *r = S(t)*, evolve according to the Stefan boundary conditions,

(5) $$\left(\varphi_{G}-\varphi_{0}\right)\frac{dR}{dt}=-D_{s}\left(\varphi_{G}\right)\left(1-\varphi_{G}\right){\left[\frac{\partial \varphi }{\partial r}\right]}_{r=R\left(t\right)},R\left(0\right)=R_{0},$$

(6) $$\frac{dS}{dt}=D_{s}\left(\varphi_{eq}\right){\left[\frac{\partial \varphi }{\partial r}\right]}_{r=S\left(t\right)},\;\;S\left(0\right)=R_{0}.$$

This model fits well with the case of a spherical initially non-porous particle for which the sharp glassy-rubbery interface progressively moves towards the center of the particle and spherical glassy inner core that progressively disappears. However, in the case of a porous particle, like the beads under investigation, we can imagine that the solvent can penetrate and diffuse inside the glassy core through the pore network and that the gelling transition occurs simultaneously both on the inside and on the external surface of the bead. Therefore, it is not possible to identify a net glassy-rubbery interface, but rather a “diffuse” interface as depicted in Figure 1. This phenomenon for porous-particles has been modeled by assuming a non-zero solvent diffusivity in the glassy phase, thus introducing a solvent concentration dependent diffusion coefficient as follows

(7) $$D_{s}\left(\varphi \right)=D_{s}^{sg}f\left(\varphi \right),f\left(\varphi \right)=\left\{\begin{array}{c} 1\;for\;\varphi \geq \varphi_{G}\\ exp\left\{-\beta \frac{\varphi -\varphi_{G}}{\varphi_{0}-\varphi_{G}}\right\}\;for\;\varphi <\varphi_{G} \end{array}\right\},$$

where *D_s_^sg^* is the solvent diffusivity in the swollen gel, assumed constant for *φ* > *φ_G_*, and *β* is a parameter controlling the decay of the diffusivity in the glassy core. The larger the porosity, the smaller *β*, the greater the ability of the solvent to penetrate in the glassy core.

The solvent transport equation and boundary conditions in highly porous particles read as

(8) $$\frac{\partial \varphi }{\partial t}=\frac{1}{r^{2}}\frac{\partial }{\partial r}\left(r^{2}D_{s}\left(\varphi \right)\left(1-\varphi \right)\frac{\partial \varphi }{\partial r}\right),0<r<S\left(t\right),\varphi \left(0\leq r\leq R_{0},t=0\right)=\varphi_{0},$$

(9) $${\left[\varphi \right]}_{r=S\left(t\right)}=\varphi_{eq},{\left[\frac{\partial \varphi }{\partial r}\right]}_{r=0}=0,$$

that are the same as Equations (3) and (4) with the basic difference that *φ(r,t)* is now defined in the entire domain *0 ≤ r ≤ S(t)* because the glassy-rubbery sharp interface *R(t)* has been removed and solvent diffusion is allowed in the glassy region with diffusion coefficient given by Equation (7). The only moving boundary is the gel-solvent interface at *r = S(t)*, evolving in time according to Equation (6).

**Figure 2:**
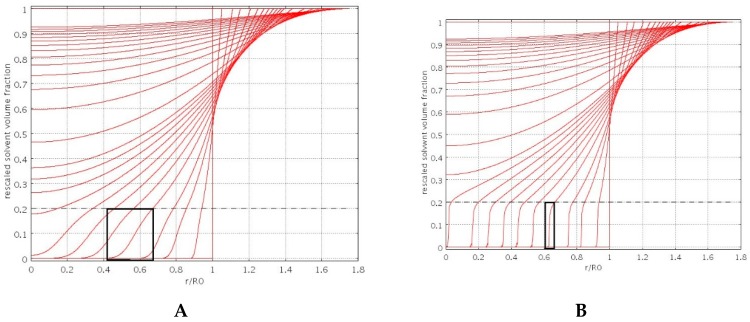
Rescaled solvent volume fraction *φ(r,t)/φ_eq_*, vs. dimensionless radius *r/R_0_* for increasing times during the swelling process. Dashed line indicates the value of *φ_G_/φ_eq_*, Black boxes highlight the thickness of the diffuse glassy-rubbery interface. (**A**) *β* = 2, porous particle; (**B**) *β* = 8, non-porous particle.

The “diffuse” interface swelling model introduces the only extra parameter *β* controlled by the bead porosity. Figure 2A,B shows the spatial behavior of the normalized solvent volume fraction *φ(r,t)/φ_eq_*, for increasing times, as obtained from the numerical solution of the swelling model Equations (6)–(9), for two different values of *β*, namely *β* = 2 (Figure 2A) and β = 8 (Figure 2B).

Solvent concentration profiles for *β* = 8 show a very rapid decay of *φ* for *φ < φ_G_*, thus exhibiting a very sharp interface, typical of a non-porous particle. On the contrary, for *β* = 2 (porous particle) concentration profiles exhibit a smoother behavior for *φ < φ_G_* because of solvent penetration in the glassy region.

The estimate of model parameters *D_s_^sg^*, *β* and *φ_eq_* can be obtained by comparing model predictions with experimental data for the temporal evolution and asymptotic value of the swelling degree *S(t)*

(10) $$S\left(t\right)=\frac{weight\;of\;absorbed\;solvent}{weight\;of\;dry\;particle}=\frac{\rho_{s}\;\int_{0}^{S\left(t\right)}\varphi \;4\pi r^{2}dr}{\rho_{b}\;(4/3)\;\pi R_{0}^{3}}$$

where *ρ_s_* and *ρ_b_* are the solvent and the freeze-dried bead densities, respectively.

The estimate of model parameters is fully addressed in Section 4.3 in connection with the analysis of swelling data in both release media SGF and SIF.

### 3.2. Drug Release Modeling of No-Interacting Drugs

If we assume no physical/chemical interaction between the drug and the polymer or the clay-polymer complex, the transport model for the drug concentration *c_d_(r,t)*, initially loaded in the glassy core, reads as

(11) $$\frac{\partial c_{d}}{\partial t}=\frac{1}{r^{2}}\frac{\partial }{\partial r}\left(r^{2}D_{d}\left(\varphi \right)\frac{\partial c_{d}}{\partial r}-r^{2}v_{r}^{s}\left(\varphi \right)c_{d}\right),0<r<S\left(t\right),c_{d}\left(0\leq r\leq R_{0},t=0\right)=c_{d}^{0},$$

(12) $${\left[c_{d}\right]}_{r=S\left(t\right)}=C_{res}\left(t\right),{\left[\frac{\partial c_{d}}{\partial r}\right]}_{r=0}=0,$$

where *v_r_^s^(φ)* is the point wise swelling velocity and *D_d_(φ)* *= D_d_^sg^(φ)f(φ)* is the drug diffusivity, modeled exactly as the solvent diffusivity *D_s_(φ)*. Indeed, *D_d_^sg^* is the drug diffusion coefficient in the swollen gel, assumed constant *for φ > φ_G_*, and *f(φ)* is the same function adopted to describe solvent penetration in the glassy core, Equation (7). Consistently with the solvent penetration model adopted, drug diffusion is allowed in the glassy core through the pore network, and the parameter β controlling diffusivity decay in the glassy core is assumed the same for the solvent and the drug.

The concentration *C_res_(t)*, entering the boundary condition at the rubbery-solvent interface, represents the drug concentration in the reservoir in which the beads are immersed for release, with volume V_res_, assumed perfectly mixed. *C_res_(t)* evolves in time according to the macroscopic balance equation accounting for drug release from swelling beads and withdrawals, modeled as an instantaneous depletion of drug concentration in the reservoir

(13) $$V_{res}\frac{dC_{res}}{dt}=N_{beads}\left(-D_{d}^{sg}{\left[\frac{\partial c_{d}}{\partial r}\right]}_{r=S\left(t\right)}4\pi S^{2}\left(t\right)\right)-\sum_{i=1}^{N_{w}^{t}}V_{w}C_{res}\left(t\right)\;\delta \left(t-t_{w}^{i}\right),$$

where *N_beads_* is the total number of swelling/releasing beads, *V_w_* is the withdrawal volume, *t_w_^i^* is the time of the *i*-th withdrawal and *N_W_^t^* is the number of withdrawals from time zero to current time *t*. The simplifying assumption of perfect sink condition is therefore replaced by the more accurate expression Equation (13) for *C_res_(t)* that, in the limit for *V_res_* */V_beads_→ ∞,* permits to recover the perfect sink condition *C_res_(t)* = 0. Equations (11)–(13) for drug transport must be solved together with Equations (6)–(9) for solvent diffusion.

**Figure 3:**
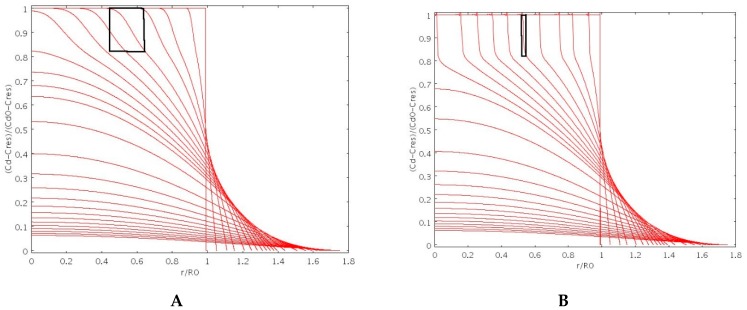
Normalized drug concentration *(c_d_−C_res_)/(c_d_^0^−C_res_)* vs. dimensionless radius *r/R_0_* for increasing times as obtained by adopting *β* = 2 ((**A**), highly porous particle) and *β* = 8 ((**B**), non-porous particle) for particle swelling and drug transport. Black boxes highlight the thickness of the diffuse glassy-rubbery interface.

Figure 3A,B shows the spatial behavior of the normalized drug concentration *(c_d_−C_res_)/(c_d_^0^−C_res_)* for increasing times as obtained by choosing *β* = 2 (porous particle) and *β* = 8 (non-porous particle) for both *D_s_(φ)* and *D_d_(φ).* We observe that, for *β* = 2 (porous particle), the drug can smoothly diffuse out of the glassy core through the pore network while, for *β* = 8 (non-porous particle), drug concentration profiles exhibit a jump at the sharp glassy-rubber interface.

The estimate of the only model parameter *D_d_^sg^* entering the drug release model is obtained by direct comparison of model predictions for *C_res_(t)* with experimental data for withdrawal drug concentrations *C_w_^i^ = C_res_(t_w_^i^)* or by direct comparison of model predictions with experimental data of the integral release curve

(14) $$\frac{M_{t}}{M_{\infty }}=\frac{V_{res}C_{res}\left(t\right)+\;\sum_{i=1}^{N_{w}^{t}}V_{w}C_{res}\left(t_{w}^{i}\right)}{V_{res}C_{res}\left(\infty \right)+\;\sum_{i=1}^{N_{w}}V_{w}C_{res}\left(t_{w}^{i}\right)},$$

where *C_res_(∞)* is the asymptotic concentration in the reservoir and *N_W_* is the total number of withdrawals made during the entire release experiment.

### 3.3. Drug Release Modeling of Interacting Drugs

If we assume a physical/chemical interaction between the drug and the clay-polymer complex we need to introduce a two-phase model, analogous to that adopted by Singh et al. (1994) [29] and by Paolicelli et al. (2017) [30] modeling drug release from hydrogels via a diffusion transport equation coupled with a sorption/desorption mechanism. The “two-phases” model adopted in the present work introduces a fraction ε of drug molecules initially bounded to the clay-polymer complex by a physical bound and a “desorption” mechanism, occurring during solvent penetration, modeled as a linear transfer rate from the adsorbed “bounded” phase to the desorbed “free” (or gel-solvent) phase where the drug is free to diffuse with diffusion coefficient *D_d_(φ),* the same adopted in Section 3.2 for non-interacting drugs.

Let us then indicate with *c_b_* and *c_d_* the drug concentrations in the bounded and free (gel-solvent) phases, respectively. Drug concentration *c_b_(r,t)* evolves in space and time according to the transport equation for the clay-polymer complex during the swelling process

(15) $$\frac{\partial c_{b}}{\partial t}=\frac{1}{r^{2}}\frac{\partial }{\partial r}\left(-r^{2}v_{r}^{s}c_{b}\right)-k_{bg}\left(\varphi \right)c_{b},\;0<r<S\left(t\right),\;c_{b}\left(r\;,t=0\right)=\epsilon c_{d}^{0},{\left[\frac{\partial c_{b}}{\partial r}\right]}_{r=0}=0,$$

but including a linear transfer rate from the bounded to the free phase *−r_b__→g_* = *k_bg_(φ)* *c_b_*, induced by solvent penetration and modeled, according to the solvent diffusion model Equation (7), as *k_bg_(φ) = k_bg_^sg^f(φ)* where *k_bg_^sg^* [1/s] is the transfer rate coefficient in the swollen gel, assumed constant for *φ > φ_G_*. Correspondingly, the transport equation for drug molecules in the free (gel) phase *c_d_(r,t)* reads as

(16) $$\frac{\partial c_{d}}{\partial t}=\frac{1}{r^{2}}\frac{\partial }{\partial r}\left(r^{2}D_{d}\left(\varphi \right)\frac{\partial c_{d}}{\partial r}-r^{2}v_{r}^{s}c_{d}\right)+k_{bg}\left(\varphi \right)c_{b},0<r<S\left(t\right),\;c_{d}\left(r,\;t=0\right)=\left(1-\epsilon \right)c_{d}^{0},$$

to be solved with the same boundary conditions Equation (12) adopted for the no-interaction model and with Equation (13) for the time evolution of the drug concentration in the reservoir *C_res_(t).*

The inverse of the transfer rate coefficient 1/*k_bg_^sg^* represents the characteristic time for the irreversible transfer of a drug molecule from the bounded to the free (gel) phase. This characteristic time *t_bg_* = 1/*k_bg_^sg^* can be compared to the characteristic drug diffusion time *t_D_ = R*_0_^2^*/D_d_^sg^* by introducing the Thiele modulus *Φ*^2^ *= t_D_/t_bg_ = k_bg_^sg^ R*_0_^2^*/D_d_^sg^* to identify the rate-controlling step.

The estimate of transport parameters, namely *D_d_^sg^*, and *k_bg_^sg^* and is obtained by direct comparison of model predictions with experimental data for withdrawal drug concentrations *C_res_(t_w_^i^)* and for the integral release curves *M_t_/M_∞_*.

### 3.4. Numerical Issues

PDE equations and boundary conditions describing the one-dimensional swelling dynamics and drug release were numerically solved using finite elements method (FEM) in Comsol Multiphysics 3.5. The convection–diffusion package was coupled with ALE (Arbitrary Lagrangian Eulerian) moving mesh. Free displacement induced by boundary velocity conditions was set. Lagrangian quadratic elements were chosen. The linear solver adopted was UMFPACK, with relative tolerance 10^−4^ and absolute tolerance 10^−7^. The number of finite elements is 10^4^ with a non-uniform mesh. Smaller elements were located close to the boundary *r = S(t)* in order to accurately compute concentration gradients controlling the velocity of the moving front.

## 4. Results and Discussion

### 4.1. Rheological Measurements

**Figure 4:**
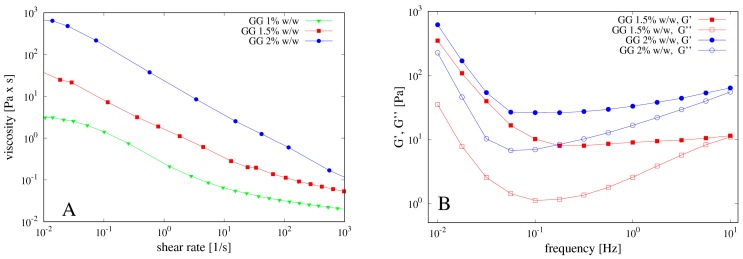
(**A**) Flow curves of gellan gum (GG) at different concentrations 1%, 1.5% and 2% *w*/*w*; (**B**) corresponding mechanical spectra.

The concentration of gellan gum (GG) used for the preparation of the beads was carefully optimized on the basis of the rheological properties of its solutions. Specifically, GG solutions at 1.0, 1.5 and 2.0% *w*/*w* were prepared at 80 °C and, after complete solubilization, cooled to 40 °C. The flow curves obtained at this temperature for the three solutions of GG are reported in Figure 4A. The viscosity of the system increases by increasing the polymer concentration and the dependence of the viscosity on the shear rate is characteristic of a pseudo-plastic macromolecular system.

Mechanical spectra were also recorded in the region of linear viscoelasticity and reported in Figure 4B. The polymeric solution at 1.0% *w*/*w* of GG showed a G’ smaller than G’’, behaving as an entanglement network (data not shown). By increasing the GG concentration, the polymeric solution evolves towards a weak gel behavior, with G’ bigger than G’’ and slightly dependent on the frequency. During the cooling, GG undergoes a series of structural changes in aqueous solution from random coiled chains to double helices, which then aggregate together thus forming a three-dimensional network. This behavior is well known as the gelation mechanism of gellan gum alone or in the presence of cations has been extensively investigated by different techniques, such as rheological, DSC and light scattering measurements and reviewed in [31].

Preliminary experiments were carried out in order to verify if the three solutions could flow through a syringe needle. GG solutions were loaded into the syringe at 40 °C. This temperature value was chosen in order to avoid any possible thermal damage when drugs are loaded inside the systems. GG concentrations greater than 1.5% *w*/*w* resulted in a highly viscous solution that caused clogging of the syringe needle, so that only the solutions at 1.0% and 1.5% *w*/*w* GG were used to prepare the beads.

The morphology of the forming beads by ionotropic gelation strongly depends on the amount of gellan gum in the initial mixture [32,33,34]. The lower the polymer content, the more deformed and irregular the beads, due to the matrix-forming function of gellan. Moreover, if the GG concentration is too low, the beads lost their spherical shape during the drying process, as already observed in [34]. In fact, both polymer and cross-linker concentrations have major effects on morphology as well as on other properties of the resulting beads, as reviewed in [10], because both concentrations affect the kinetic of the crosslinking process.

Our preliminary tests showed that the polymer concentration represents the main factor controlling the morphology of the beads. For GG concentrations lower than 1.5% *w*/*w*, the solution was unable to produce stable spherical beads, most likely because the low viscosity of the solution does not allow to keep the spherical shape of the drops when in contact with the cross-linking solution. For this reason, concentrations of GG below 1% *w*/*w* were not further investigated and the GG concentration of 1.5% *w*/*w* was chosen (see Section 4.2).

**Figure 5:**
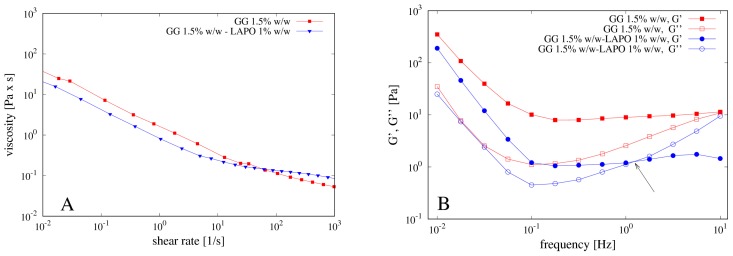
(**A**) Flow curves of GG solution with and without laponite and (**B**) mechanical spectra of the same solutions. Arrow indicates the inversion point G’’ ≈ G’ at a frequency of about 1 Hz for the mechanical spectra of the GG solution including laponite.

The effect of clay on the rheological properties of GG was also investigated. To this end, 1.0% *w*/*w* of laponite was added to the GG solution at 1.5% *w*/*w*. Flow curves and mechanical spectra performed on the polymeric solution containing the clay are shown in Figure 5A,B.

The presence of laponite induces a small decrease of viscosity of the polymeric solution as well as of the G’ value, most likely because the clay interacts with the gellan chains, thus partially destroying the double helix structure and causing the formation of a weaker gel. Indeed, the mechanical spectrum of the GG solution shows a weak gel behavior with the modulus G’ higher than G’’ in the entire range of frequencies analyzed. On the contrary, the GG solution with laponite shows an intersection point of the G’ and the G’’ curves at frequency of about 1 Hz, thus behaving as a solution (with G’’ > G’) for higher values of the applied frequency.

### 4.2. Beads Preparation

Based on the rheological properties of its solutions, different GG beads were prepared dropping GG solution at 1% *w*/*w* and 1.5% *w*/*w* into CaCl_2_ solutions at different concentrations in order to evaluate the effect of the cross-linking agent on the properties of the resulting beads.

The crosslinking process involves calcium ions and carboxylic groups of D-glucuronic acid of GG. More specifically, every calcium ion can electrostatically interact with two carboxylic groups; therefore, it interacts with two d-glucuronic acids of two different repetitive units of the polymer.

The crosslinking concentration always exceeded the concentration of the polymer as molar ratios GG:Ca^2+^ of 1:5, 1:7.5, 1:10 and 1:15 mol:mol were investigated. Regular and spherical beads were obtained with GG concentration 1.5% *w*/*w* for 1:5 and 1:10 GG:Ca^2+^ molar ratios, corresponding to CaCl_2_ concentrations of 0.3% *w*/*w* and 0.6% *w*/*w*, respectively. Concentrations of CaCl_2_ < 0.3% *w*/*w* have not produced stable and spherical beads. GG solution 1.0% *w*/*w* gave irregular beads even for higher GG:Ca^2+^ molar ratios 1:7 and 1:15. Based on these results, concentrations of GG below 1% *w*/*w* were not further investigated and the GG concentration of 1.5% *w*/*w* with CaCl_2_ concentrations of 0.3% *w*/*w* and 0.6% *w*/*w* were adopted because these concentrations did not cause clogging of the syringe needle and produced regular and spherical beads. Further formulations were prepared by adding laponite to GG solution before beads formation. In this case, the beads were formed using the GG solution 1.5% *w*/*w* with laponite 1% *w*/*w* and with the lower concentration 0.3% *w*/*w* of CaCl_2_, which was chosen by considering that the clay is able to act as cross-linker itself, thus contributing to the polymeric network formation.

The beads were recovered by filtration and characterized immediately after preparation in their fresh form and after the freeze-drying process. Specifically, they were observed at the optical microscope and their diameters measured and reported in Table 1.

**Figure 6:**
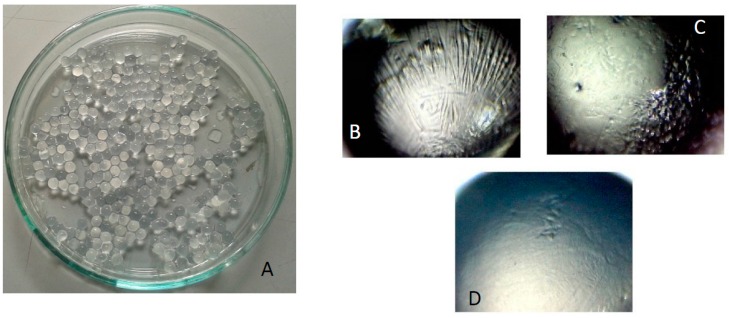
(**A**) Pictures of beads of GG/LAPO/Ca 0.3%; pictures of beads at the optical microscope; (**B**) GG/Ca 0.3%; (**C**) GG/Ca 0.6%; (**D**) GG/LAPO/Ca 0.3%.

Different formulations lead to beads with different dimensions: the cross-linker concentration does not influence significantly the particle diameter, whereas the presence of laponite leads to an increase of the particle diameter. For all formulations, the bead population appear homogeneous and with spherical shape (see Figure 6A) characterized by an aspect ratio of about 1.02. The beads containing laponite (Figure 6D) have a smoother and regular surface with respect to the other ones (Figure 6B,C which differ for the CaCl_2_, concentration), most likely because the clay, acting as filler, increases the particle surface compactness.

Particle density after freeze-drying ρ_b_ is extremely low and comparable for all formulations. Specifically, *ρ_b_* = 0.109 ± 0.02 g/cm^3^ for GG/Ca 0.3% and *ρ_b_* = 0.0926 ± 0.02 g/cm^3^ for GG/LAPO/Ca 0.3%.

### 4.3. Swelling Experiments

A crucial property of the polymeric beads is the ability to swell in aqueous environments. The results of swelling experiments are reported in Table 2 in terms of the swelling degree at equilibrium *S_eq_* (after 24 h).

In general, the swelling degree decreases in both swelling media as the amount of cross-linker is increased. The significant differences observed in swelling degree values in SGF and SIF are related to the nature of GG. The carboxylic groups of GG exist in a protonated form in HCl. This allows the network chains to stay closer to each other, resulting in a smaller swelling degree in acid medium. The beads in SIF exhibit a larger swelling degree as the carboxylic groups are deprotonated, resulting in a repulsion effect between network chains.

The presence of laponite causes a remarkable decrease of the equilibrium value *S_eq_* as already observed in [17] dealing with beads made of pH sensitive laponite/alginate/CaCl_2_ hybrid hydrogel. In agreement with experimental findings reported in [17], the effect of clay is not only to decrease the equilibrium swelling degree but also to reduce the solvent diffusion coefficient *D_s_^sg^* in both media, as reported in Table 2. In agreement with swelling degrees at equilibrium, Table 2 also shows that *D_s_^sg^* is larger for SIF than for SGF for particles with and without clay. The values of *D_s_^sg^* reported in Table 2 for different beads and different media are obtained from the best-fit of experimental data for the time evolution of the swelling degree *S(t)* with the swelling model developed in Section 3.1.

**Figure 7:**
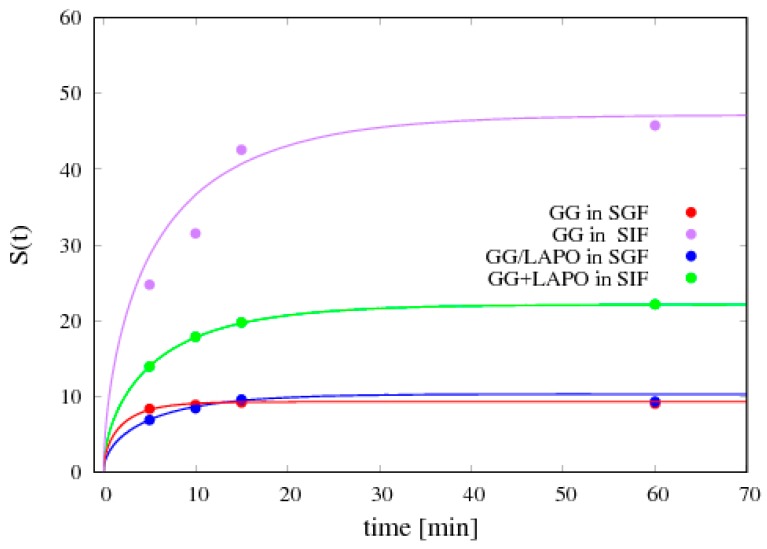
Comparison between the swelling model predictions (continuous lines) and experimental data (points) for the temporal evolution of the swelling degree *S(t)* for two different bead formulations (GG/Ca 0.3% and GG/LAPO/Ca 0.3%) and two different swelling media (SGF and SIF). The best fit values for the solvent diffusion coefficient in the swollen gel *D_s_^sg^* are reported in Table 2.

Figure 7 shows the comparison between experimental data for *S(t)* and the swelling model predictions where:

(1) the parameter *β* has been set to the value *β* = 2 in order to account for the large porosity/small density of beads;
(2) the solvent volume fraction (SGF or SIF) at equilibrium *φ_eq_* is directly estimated from the equilibrium swelling degree *S_eq_* as
  (17) $$\varphi_{eq}=\frac{x_{eq}/\rho_{s}}{\left(1-x_{eq}\right)/\rho_{b}+x_{eq}/\rho_{s}},\;x_{eq}=1-1/\left(1+S_{eq}\right),$$
  where *x_eq_* is the solvent weight fraction at equilibrium and *ρ_b_* is the dry particle density;
(3) *φ_G_* has been set to *φ_G_* = 0.1*φ_eq_*, given the ease of beads re-hydration after freeze-drying.

**Figure 8:**
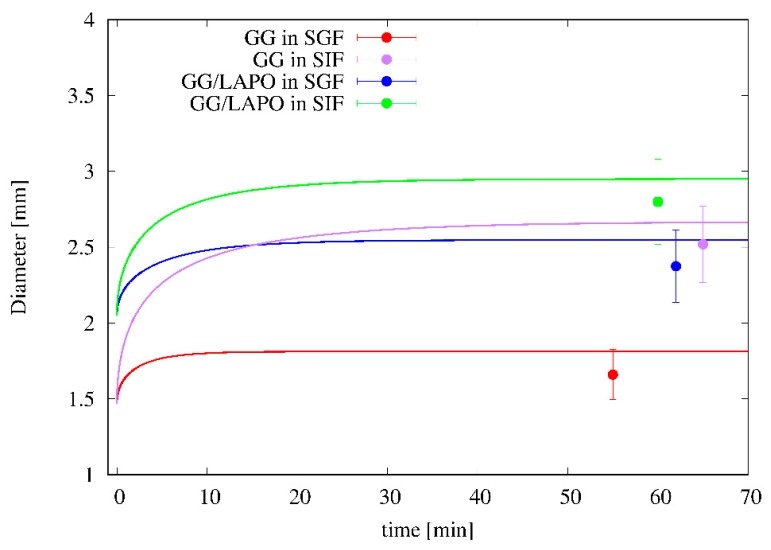
Comparison between the swelling model predictions (continuous lines) and experimental data (points) for the temporal evolution of the particle diameter for two different bead formulations (GG/Ca 0.3% and GG/LAPO/Ca 0.3%) and two different swelling media (SGF and SIF).

Figure 8 shows the time evolution of particle diameter as predicted by the swelling model and the satisfactory agreement with experimental data for particle diameter at equilibrium. It can be observed that dry particles, given the high porosity and the small density, are able to absorb a large amount of solvent, e.g., about 50 times their initial weight for GG/Ca 0.3% formulation in SIF, while the diameter at equilibrium at the most doubles its initial value. The swelling model, being able to account for the high particle porosity, furnishes a reliable forecasting estimate of the equilibrium diameter and of the time-scale for reaching equilibrium conditions, approximately 15–20 min for all the different formulations in the two media.

It is important to point out that, even if the increase in the particle diameter is not so large, it is however extremely important to adopt a release model that takes into account the swelling and the variation of the diameter of the particle over time, as the estimate of the effective drug diffusivity is based mainly on the analysis of release curves at short time scales and is strongly influenced by the variation of the diffusional lengths. Adopting a fixed boundary model leads to an error of the drug diffusion coefficient at least of a factor (*d_eq_*/*d*_0_)^2^ where *d_eq_* and *d*_0_ are the equilibrium and initial particle diameter, respectively.

### 4.4. Entrapment Efficiency

Two model drugs of different molecular weights and dimensions were loaded into the beads, namely theophylline (MW 180, van der Waals radius 3.7 Å, aqueous solubility 8.3 mg/mL; pKa 8.6 [35]) and vitamin B12 (MW 1356, van der Waals radius 21 Å, aqueous solubility 10–33 mg/mL, pKa = 3.28 [36]) in order to verify the possible use of freeze-dried beads for the oral administration of drugs.

Table 3 reports the entrapment efficiency of the two drug molecules into two different bead formulations, GG/Ca 0.3% *w*/*w* and GG/LAPO/Ca 0.3% *w*/*w*. It is evident that the entrapment efficiency is influenced by both the bead structure and the steric hindrance of the loaded molecule. Indeed, theophylline, smaller than vitamin B12, is less retained by both bead formulations, whereas the presence of laponite increases the entrapment efficiency of both drug molecules. This will reflect in drug-release data analyzed in the next section. The fact that the presence of laponite increases the drug entrapment efficiency has been already observed in [17] for methylene blue loaded laponite/alginate beads.

### 4.5. Release Data Analysis

#### 4.5.1. Theophylline Release

**Figure 9:**
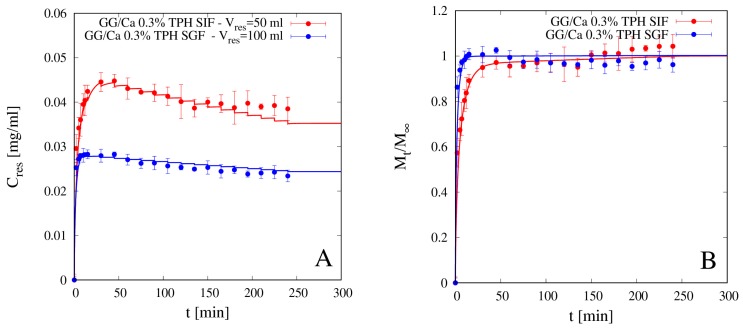
Differential (**A**) and integral release curves (**B**) for theophylline (TPH) from beads GG/Ca 0.3% *w*/*w* without laponite in SGF and SIF. Continuous lines represent model predictions Equations (11)–(13). Estimated values of the TPH effective diffusivity *D_d_^sg^* are reported in Table 4.

We preliminary analyzed release data of theophylline from beads GG/Ca 0.3% *w*/*w* without laponite in both media SGF and SIF. Release data are shown in Figure 9A,B in terms of the withdrawal concentration *C_res_ (t_w_^i^)*, from now on referred to as differential release curve, and of the integral release curve *M_t_/M_∞_*.

It can be observed that integral release curves exhibit typical Fickian behavior in both media and any type of physical/chemical drug/polymer interaction can be excluded. Continuous lines represent theoretical predictions obtained with the no-drug-polymer interaction model Equations (11)–(13) developed in Section 3.2, numerically solved together with the swelling model Equations (6)–(9) for which the parameter values, specifically *D_s_^sg^* and *β*, reported in Section 4.3, were estimated from independent swelling measurements. The only best-fit parameter entering the drug release model is the theophylline diffusivity in the swollen gel *D_d_^sg^*, whose values are reported in Table 4 for both media.

It is important to point out that, for an accurate estimate of *D_d_^sg^*, the model best-fit must be performed on differential experimental data of *C_res_ (t_w_^i^)*, instead of on the integral release curve *M_t_/M_∞_*, *C_res_ (t_w_^i^)* data being more sensitive to *D_d_^sg^* both in the initial phase of rapid concentration rise and in the subsequent phase where the effect of withdrawals become significant and not negligible.

The finite volume *V_res_* of the reservoir is explicitly taken into account in model formulation, as well as the withdrawals, as can be observed from the sawtooth behavior of the differential release model curve. In fact, smaller values of *V_res_*, which do not guarantee the perfect sink condition, are to be preferred as they ensure better mixing and greater uniformity of drug concentration in the reservoir. Imperfect mixing is difficult to model and leads to a withdrawal drug concentration that may depend on the withdrawal point. On the contrary, the non-negligible drug concentration in the perfectly mixed reservoir (*C_res_* > 0, no sink condition) can be easily modeled by means of a macroscopic balance equation (see Equation (13)). The only requirement is that *V_res_* must be greater than a minimum value that guarantees that the maximum value attained by *C_res_(t)* during the release experiment is significantly lower than drug solubility in the release medium. This condition is always fulfilled in our release experiments for both drugs and for all *V_res_* analyzed.

From integral release data shown in Figure 9B and diffusivity values reported in Table 4 it can be readily observed that theophylline release from beads without clay, despite the larger degree of swelling in SIF than in SGF, is faster in SGF than in SIF. Theophylline effective diffusivity in the bead swollen in SGF is about half the value in aqueous solution *D_TPH_* ≈ 8.2 × 10^−10^ m^2^/s, while it reduces to a quarter of *D_TPH_* in the bead swollen in SIF. This can be explained in terms of the screening effect of the COO^–^ groups of GG by the ions in SGF solution, thus reducing the possible interaction between theophylline and the charged polymer. A similar phenomenon has been observed by Coviello et al. (1999) [37] for theophylline in sclerox, a polycarboxylated derivative of scleroglucan.

In [37] the authors reported that theophylline diffusion rate in sclerox, without crosslinker, was increased in acid medium with respect to that in SIF, while a diffusion rate lower in acid medium than in SIF was observed in sclerox in the presence of alkane dihalides as crosslinker. We observed the same inversion phenomenon from release data of theophylline in GG beads with and without laponite, acting as a crosslinker as supported by swelling data.

**Figure 10:**
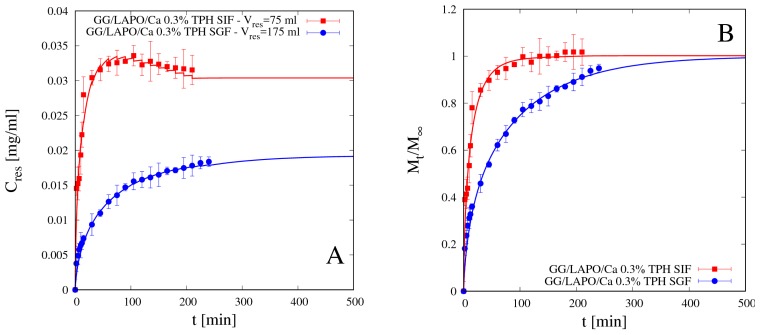
Differential (**A**) and integral release curves (**B**) for theophylline (TPH) from beads GG/LAPO/Ca 0.3% with laponite in SGF and SIF. Continuous lines represent model predictions from Equations (11)–(13). Estimated values of the TPH diffusivity *D_d_^sg^* are reported in Table 4.

Figure 10A,B shows release data for theophylline from beads GG/LAPO/Ca 0.3% in SGF and SIF and the comparison with model predictions with the corresponding effective diffusion coefficients *D_d_^sg^* reported in Table 4. In the presence of clay, theophylline diffusivity reduces by an order of magnitude with respect to *D_TPH_* in both media, but it is significantly smaller in SGF than in SIF. In this case, diffusivity values are in agreement with swelling data, *S_eq_* being significantly larger in SIF than in SGF for GG beads including laponite (see Table 2). Therefore, the screening effect of the COO^–^ groups of GG by the ions in SGF solution, although still present, is balanced and overcome by the reduced mesh size of the network, this last observation being supported by swelling data in Figure 7 and Table 2.

This observation is in agreement with experimental findings reported in [17] focusing on drug release (methylene blue) from beads made of pH sensitive laponite/alginate/CaCl_2_ hybrid hydrogel. These authors observed that the presence of laponite induced a significant slowing down of the release kinetic of hydrophilic drugs especially in acid medium.

Reliability and accuracy of the experimental release curves above presented, as well the predictive ability of the release model proposed, are confirmed by the analysis of gastrointestinal release data, i.e., release data obtained by immersing the beads sequentially first in SGF for 120 min and subsequently in SIF for 120 min, thus simulating the gastrointestinal release in-vitro.

**Figure 11:**
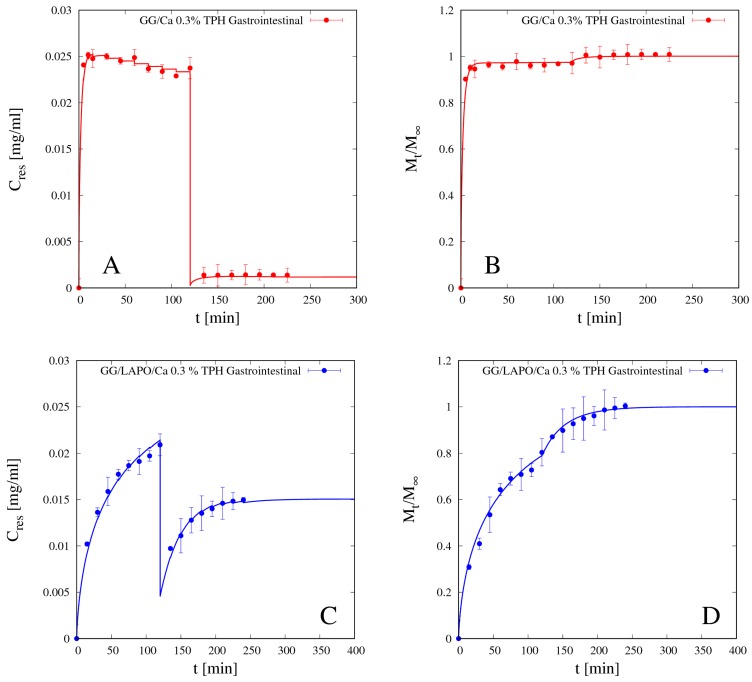
Gastrointestinal differential (**A**,**C**) and integral release curves (**B**,**D**) for theophylline (TPH) from beads GG/Ca 0.3% without laponite (**A**,**B**) and GG/LAPO/Ca 0.3% with laponite (**C**,**D**), *p* < 0.001. Continuous lines represent model predictions from Equations (11)–(13) with TPH diffusivity *D_d_^sg^* reported in Table 4.

Figure 11A–D shows the differential (A and C) and the integral (B and D) gastrointestinal release curves of theophylline from beads GG/Ca 0.3% (A,B) without laponite and GG/LAPO/Ca 0.3% with laponite (C,D). Continuous lines show the excellent agreement between experimental data and theoretical curves obtained from the release model used in a fully predictive way, by making use of the TPH diffusivity values *D_d_^sg^* previously estimated and reported in Table 4.

It can be observed that TPH release from beads without laponite is so fast in SGF that 95% of the drug is released in acid medium in the first 120 min. On the contrary, the presence of laponite significantly slows down TPH release in SGF, so that about 70% of drug is released in acid medium, and the remaining 30% is slowly released in the intestinal tract. We conclude that bead formulation including laponite represents a release medium capable of supporting the controlled release of a small non-interacting drug as theophylline.

#### 4.5.2. Vitamin B12 Release

**Figure 12:**
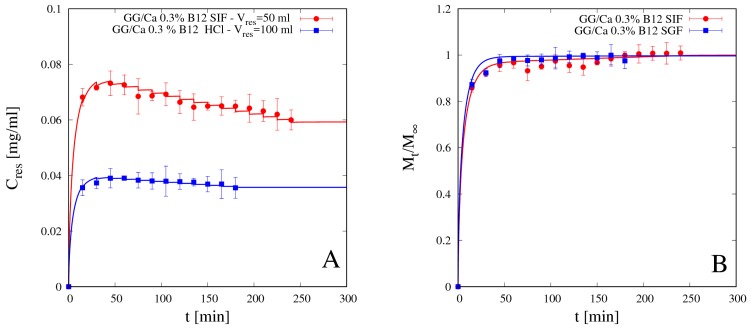
Differential (**A**) and integral release curves (**B**) for vitamin B12 from beads GG/Ca 0.3% without laponite in SGF and SIF. Continuous lines represent model predictions from Equations (11)–(13). Estimated values of the B12 diffusivity *D_d_^sg^* are reported in Table 5.

We preliminary analyzed release data of vitamin B12 from beads GG/Ca 0.3% without laponite in both media SGF and SIF. Differential and integral release data are shown in Figure 12A,B respectively. Additionally, for vitamin B12, as for theophylline, integral release curves from bead formulation without laponite exhibit typical Fickian behavior in both media. Continuous lines represent theoretical predictions obtained with the no-drug-polymer interaction model in Equations (11)–(13) developed in Section 3.2. B12 diffusivity values *D_d_^sg^* in SGF and SIF are reported in Table 5.

It can be observed that B12 diffusivities in both media are: (1) smaller than the corresponding ones for theophylline, as expected given the larger dimension of vitamin B12 and the bigger steric hindrance; (2) comparable in SIF and SGF, despite the larger differences between swelling degrees, as the screening effect in acid medium compensates the reduced mesh size of the network. Moreover, vitamin B12 diffusivity in SIF is in quantitative agreement with diffusivity value *D_B12_* ≈ 2.1 × 10^−10^ m^2^/s reported in [38] for vitamin B12 in scleroglucan/borax hydrogel swollen in distilled water (pH 5.4).

**Figure 13:**
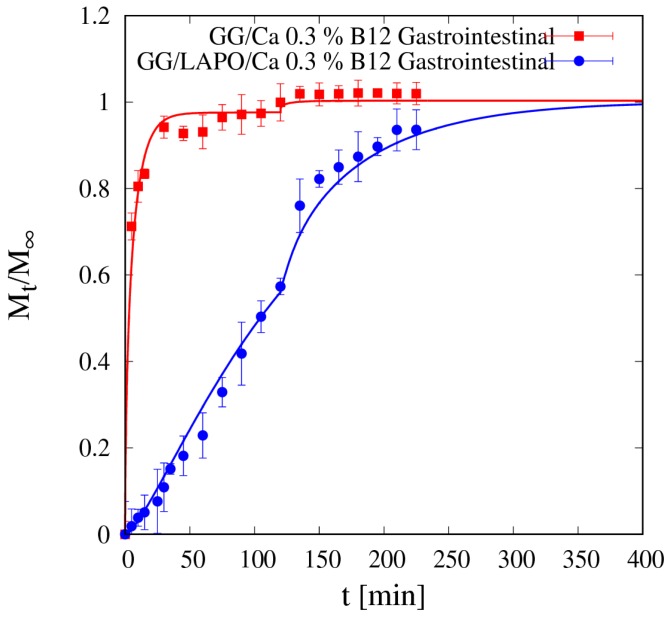
Gastrointestinal integral release curve for vitamin B12 from beads GG/Ca 0.3% with and without laponite (*p* < 0.001). Continuous lines represent model predictions for the Fickian release model from Equations (11)–(13) and for the two-phase release model from Equations (13)–(16) with model parameters reported in Table 5.

Like for theophylline, vitamin B12 release from beads without clay in SGF is fast enough so that the gastrointestinal release data shown in Figure 13 present an almost complete drug release in the gastric tract (98% of drug released after 120 min in SGF) and almost no drug is left to be released in the intestinal tract. Therefore, the bead formulation without laponite is not suitable for controlled release of a medium/large non-interacting drug molecule.

**Figure 14:**
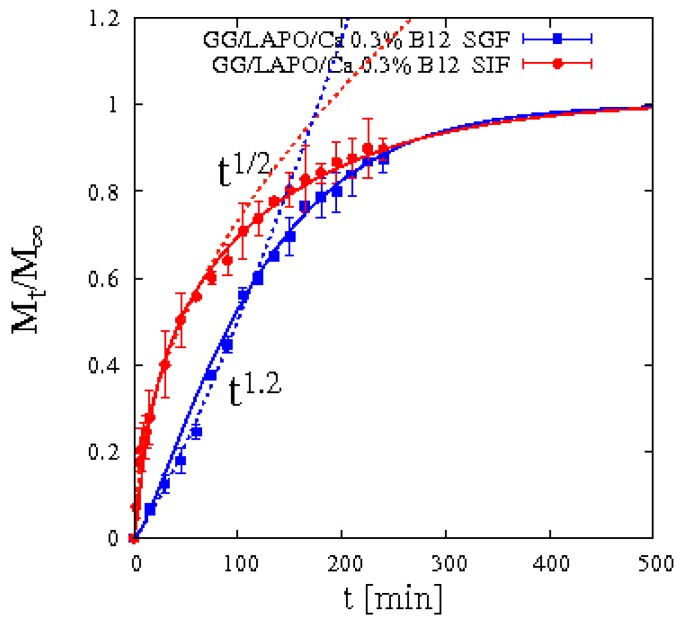
Integral release curve for vitamin B12 from beads GG/LAPO/Ca 0.3% with laponite in SGF and SIF. Dotted curves highlight the Fickian and non-Fickian behaviors in SIF and in SGF, respectively. Continuous lines represent model predictions from Equations (13)–(16).

A completely different scenario opens up when laponite is included in bead formulation and vitamin B12 release data are analyzed. Figure 14 shows integral release data of vitamin B12 from beads GG/LAPO/Ca 0.3% including laponite in SIF and SGF. It can be readily observed that drug release in SGF exhibits non-Fickian behavior *M_t_/M_∞_* ≈ *t^n^* with an exponent *n* = 1.2 much bigger than ½.

This is a clear symptom that an interaction occurs between vitamin B12 and the polymer/clay complex. This interaction is strongly weakened by the enlarged mesh size of the network induced by matrix swelling in SIF while it is stronger and sustained by the reduced swelling in SGF.

For this reason, we adopted the two-phase model developed in Section 3.3 describing drug/clay-polymer interaction in terms of a linear transfer rate from a bounded phase, in which drug molecules are initially entrapped, to a gel phase in which drug molecules are free to diffuse and exit the swelling bead. Figure 14 shows good agreement between integral release data and the two-phase model predictions Equations (13)–(16). Best-fit values of vitamin B12 diffusivity *D_d_^sg^* and transfer rate constant *k_bg_^sg^* in SIF and SGF are reported in Table 5.

The transfer rate coefficient *k_bg_^sg^* for vitamin B12 in SGF is one order of magnitude smaller than that in SIF. The diffusivity D_d_^sg^ is smaller in SGF than in SIF.

By analyzing these findings in terms of the Thiele modulus *Φ*^2^ (introduced in Section 3.3), we observe that *Φ*^2^ for vitamin B12 in GG/LAPO/Ca 0.3% attains the following values: *Φ*^2^ ≈ 80 in SIF and *Φ*^2^ ≈ 9 in SGF. Therefore, *Φ*^2^ in SGF is one order of magnitude smaller than *Φ*^2^ in SIF. This quantitatively explains why drug-polymer/clay complex interaction leads to a strong non-Fickian behavior in SGF, characterized by a slower release at short time scale because the diffusion time-scale is comparable to the transfer time scale, while a Fickian behavior is observed for vitamin B12 in SIF because diffusion is definitely the rate controlling step.

Diffusivity values *D_d_^sg^* for vitamin B12 are actually much lower in the beads with laponite in both media, and this, together with drug interaction with the polymer/clay complex, reflects in the gastrointestinal release curve shown in Figure 13 together with the corresponding vitamin B12 release curve from beads without laponite. The continuous blue curve represents the two-phase model prediction, from Equations (13)–(16), of the gastrointestinal release without any adjustable parameters.

In the presence of laponite, only 60% of the loaded vitamin B12 is released in the gastric tract (first 120 min), and also the remaining 40% is slowly released in the intestinal tract, as the complete release requires about 280 min in SIF medium. Therefore, the bead formulation including laponite is suitable for sustained release of a medium/large interacting drug molecule.

## 5. Conclusions

Gellan gum, a natural polysaccharide, was employed together with calcium chloride, selected as cross-linker, in order to prepare beads using the ionotropic gelation method. Laponite, a synthetic clay, was also included in the formulations. Stable and spherical beads (aspect ratio ≈ 1.02) were obtained from GG solutions (GG 0.15 % *w*/*w*) and GG/laponite solutions (GG 0.15% *w*/*w*, laponite 0.1% *w*/*w*) with CaCl_2_ 0.3% *w*/*w*. Gellan gum beads including laponite have shown a smoother and regular surface and a larger diameter, namely *d*_0_ ≈ 2.8 mm and *d*_0_ ≈ 2.1 mm before and after freeze-drying, respectively. The ability to swell in different media mimicking biological fluids, namely SGF and SIF, was investigated. The bead swelling degree at equilibrium was lower in SGF than in SIF and further reduced in the presence of laponite.

Two model drugs, theophylline and vitamin B12, having different molecular weight and steric hindrance, were loaded into different bead formulations. The presence of laponite in the bead formulation increased the drug entrapment efficiency for both model drugs. Sustained release of both model drugs was obtained from beads including laponite, as a small fraction of the incorporated drugs was released in the gastric medium. This suggests that laponite may be an effective additive in the development of GG beads for sustained release of drugs. Better results in terms of sustained release were obtained for vitamin B12 as it exhibited a significant interaction with the clay/polymer composites in SGF. In the absence of laponite, both drugs were almost completely released in the first two hours of residence in SGF.

The swelling model with “diffuse interface” and the corresponding drug release models developed for polymer/clay beads no-interacting and interacting drugs have proved to be able to correctly describe all the phenomena experimentally observed and to furnish reliable drug diffusivity values in agreement with literature data for the same drugs in similar physical hydrogels.

## Figures and Tables

**Table 1:** Diameter values of different bead formulations before and after freeze-drying.

Beads Formulation	Beads Diameter (mm ± SD)	Freeze-Dried Beads Diameter (mm ± SD)
GG/Ca 0.3%	2.41 ± 0.06	1.63 ± 0.03
GG/Ca 0.6%	2.44 ± 0.07	1.56 ± 0.09
GG/LAPO/Ca 0.3%	2.79 ± 0.11	2.06 ± 0.08

**Table 2:** Swelling degree at equilibrium *S_eq_* (measured after 24 h) and effective solvent diffusion coefficient *D_s_^sg^* of different bead formulations in Simulated Gastric Fluid (SGF; HCl 0.1 M) and Simulated Intestinal Fluid (SIF; phosphate buffer 0.044 M, pH 7.4).

Beads Formulation	*S_eq_* in SGF	*S_eq_* in SIF	*D_s_^sg^* in SGF [m^2^/s]	*D_s_^sg^* in SIF [m^2^/s]
GG/Ca 0.3%	9.08 ± 0.3	45.80 ± 0.9	(8.8 ± 0.3) × 10^−10^	(1.5 ± 0.1) × 10^−9^
GG/Ca 0.6%	8.97 ± 0.2	25.44 ± 0.6	-	-
GG/LAPO/Ca 0.3%	9.14 ± 0.3	20.60 ± 0.3	(6.5 ± 0.3) × 10^−10^	(1.1 ± 0.1) × 10^−9^

**Table 3:** Entrapment efficiency of two model drug molecules, theophylline and vitamin B12.

Beads	Drug Molecule	Entrapment Efficiency (%)
GG/Ca 0.3%	Vitamin B12	53.62
GG/LAPO/Ca 0.3%	Vitamin B12	61.26
GG/Ca 0.3%	Theophylline	20.26
GG/LAPO/Ca 0.3%	Theophylline	36.49

**Table 4:** Theophylline diffusivity in the swollen gel *D_d_^sg^* for two different bead formulations (with and without laponite) and two different media SGF and SIF.

Beads	*D_d_^sg^* in SGF [m^2^/s]	*D_d_^sg^* in SIF [m^2^/s]
GG/Ca 0.3%	(4.26 ± 0.15) × 10^−10^	(2.62 ± 0.2) × 10^−10^
GG/LAPO/Ca 0.3%	(2.73 ± 0.3) × 10^−11^	(1.43 ± 0.1) × 10^−10^

**Table 5:** Vitamin B12 diffusivity in the swollen gel *D_d_^sg^* and transfer rate coefficient *k_bg_^sg^* for two different bead formulations (with and without laponite) and two different media SGF and SIF.

Beads	*D_d_^sg^* in SGF [m^2^/s]	*D_d_^sg^* in SIF [m^2^/s]	*k_bg_^sg^* in SGF [1/s]	*k_bg_^sg^* in SIF [1/s]
GG/Ca 0.3%	(1.53 ± 0.15) × 10^−10^	(1.85 ± 0.15) × 10^−10^	-	-
GG/LAPO/Ca 0.3%	(2.87 ± 0.1) × 10^−11^	(5.33 ± 0.1) × 10^−11^	2.35 × 10^−4^	4.12 × 10^−3^
